# Professor Dr. Bangalore Krishnaswamy Venkataraman (1923–2018): An Icon and Pioneer of Oral Medicine in India

**DOI:** 10.7759/cureus.70837

**Published:** 2024-10-04

**Authors:** Ramachandra Reddy Gowda Venkatesha, Karthik Rajaram Mohan, Sabitha Gokulraj, Reethika Rathan, Kumar Appusamy

**Affiliations:** 1 Oral Medicine and Radiology, Vinayaka Mission's Sankarachariyar Dental College, Vinayaka Mission's Research Foundation (Deemed to be University), Salem, IND

**Keywords:** diagnostic oral sciences, historical vignette, indian academy of oral medicine and radiology, legacy, oral medicine and radiology

## Abstract

Professor Dr. Bangalore Krishnaswamy Venkataraman, a pioneer in oral medicine, significantly shaped dental education and practice in India and beyond. Rising from humble beginnings, he became a leading authority in dentistry, particularly in oral medicine and radiology. Dr. B.K. Venkataraman's pioneering work in oral medicine has left an indelible mark on dental education and practice in India and beyond. His enduring impact on dental medicine and education in India is celebrated through various commemorations, including conferences and educational initiatives. Even after retirement, he continued to influence the field as a professor and examiner in international institutions. Dr. B.K. Venkataraman's contributions have left a lasting mark, inspiring future generations in dentistry.

## Introduction and background

Professor Dr. Bangalore Krishnaswamy Venkataraman was a prominent figure in the field of oral medicine and radiology in India, known for his significant contributions to dental education and practice [1]. Professor Dr. B.K. Venkataraman was born on October 26, 1923, in Hiriyur, Chitradurga district, to Dr. B.G. Krishnaswamy and Shrimathi. Mangayarkarasi. Professor Dr. B.K. Venkataraman dedicated his life to advancing the specialty and influencing generations of dental professionals. He was married to Shrimathi. V. Bhuvaneshwari on February 3, 1956. His legacy continues not only in dentistry but also through his illustrious son, Professor Dr. B.V. Murali Mohan, a senior consultant pulmonologist and physician and academic director of the Department of Internal Medicine and Pulmonology, Narayana Health-Mazumdar Shaw Medical Center, Bangalore, and his wife, Professor Dr. Pratima Murthy who is the director at the National Institute of Mental Health and Neurosciences (NIMHANS), Bangalore [1]. He dedicated his life to advancing the specialty and influencing generations of dental professionals. Dr. B.K. Venkataraman grew up in Mysore, where he was instilled with values of service and discipline from a young age. He completed his medical education at Mysore Medical College, which laid the groundwork for his future endeavors in oral medicine [2]. Dr. B. K. Venkataraman's humble beginnings significantly influenced his career in dentistry, shaping his values, work ethic, and commitment to the field [2].

The aspects of the early life of Dr. B. K. Venkataraman significantly impacted his professional journey [3]. Dr. B.K. Venkataraman imbibed the values of discipline and service through his family, which guided his education and patient care approach. This foundation instilled a profound sense of responsibility and dedication to his work [3]. Dr. B. K. Venkataraman's father, Dr. B.G. Krishnaswamy, a medical officer whose career and values significantly influenced Dr. B.K. Venkataraman's path in medicine and dentistry. His father's commitment to medical service and his view of alleviating suffering were instilled in the mind of Dr. B.K. Venkataraman [4]. This foundational influence not only guided Dr. B.K. Venkataraman's dedication to advancing the field of oral medicine in India but also shaped his contributions to dental education and patient care throughout his career [4]. His humble background fostered a deep appreciation for education and knowledge [5]. This commitment was evident in his later efforts to enhance dental education by introducing oral medicine as a formal subject in dental curricula, thereby improving the standards of education in the field [5]. This foundation instilled a sense of responsibility and dedication to his work [5, 6].

**Figure 1:**
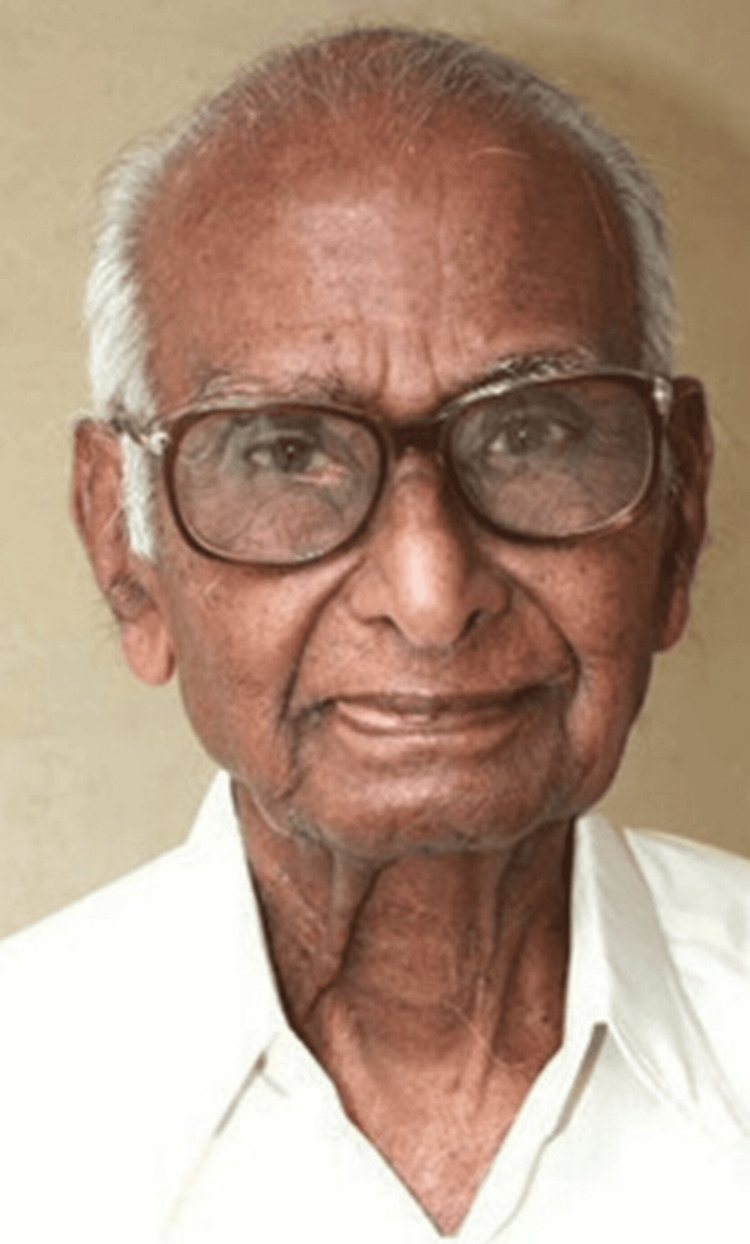
Professor Dr. B. K. Venkataraman Image source: [3] Image courtesy: Professor Dr. K. S. Nagesh, Former Faculty Government Dental College, Bangalore, Founder Professor and Head of the Department and Principal, D.A. Pandu Memorial R.V. Dental College, Bangalore. This work is licensed under Creative Commons Attribution-Noncommercial 4.0 International.

Dr. B.K. Venkataraman's parents passed away at an early age, leaving a large and young family with little support. However, his maternal grandmother took on the responsibility of caring for them. She imbibed in them the virtues of hard work, discipline, respect for education, and a strong sense of family values. His difficulties in his early childhood also gave Dr. B.K. Venkataraman has a deep sense of concern and empathy for students who come from rural and small-town backgrounds and humble circumstances (Figure 1) [7, 8].

Educational and professional journey

**Figure 2:**
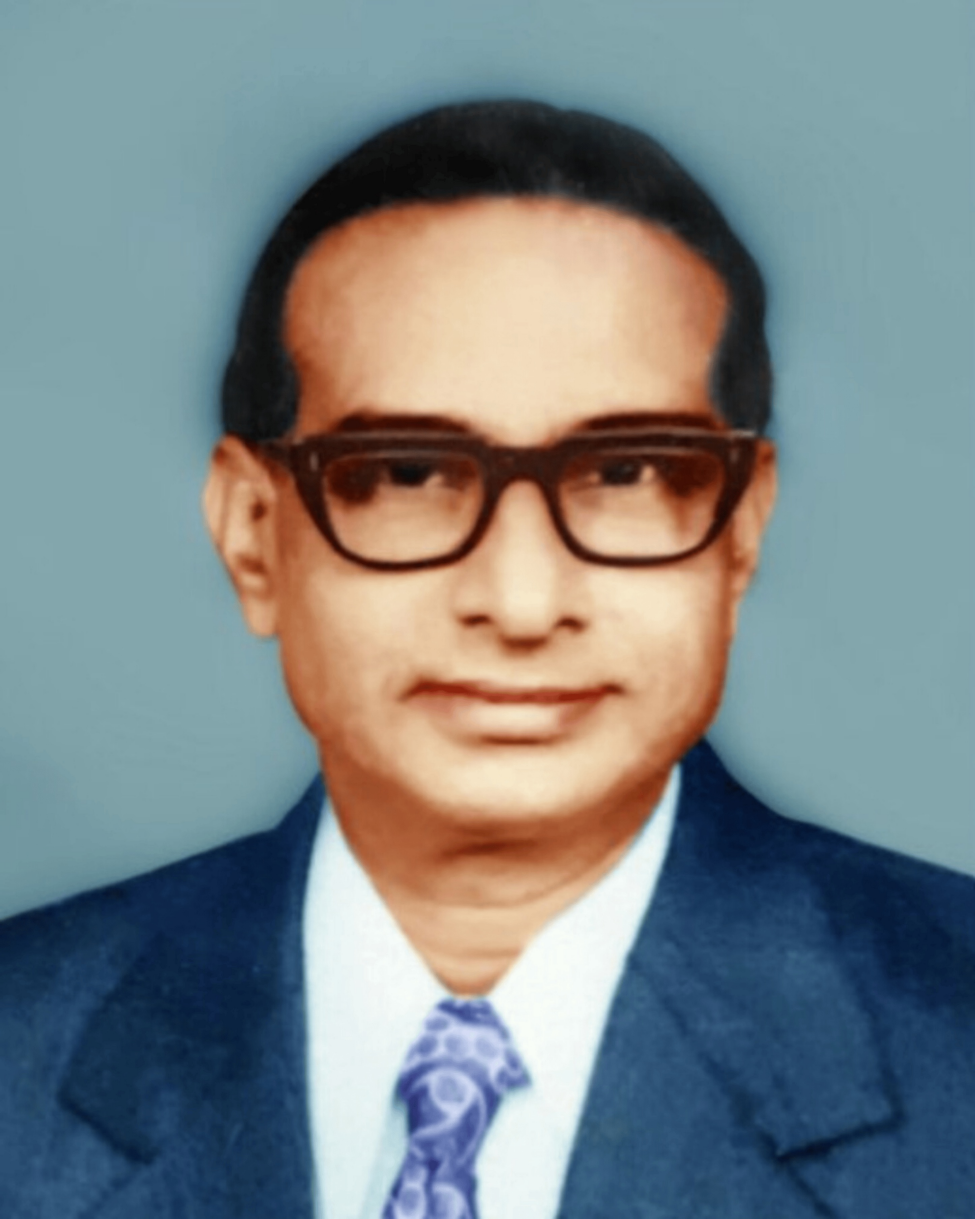
Professor Dr. B.K. Venkataraman, the father of oral medicine in India Image courtesy: Professor Dr. B.V. Murali Mohan, Senior Consultant Pulmonologist and Physician, Academic Director in the Department of Internal Medicine and Pulmonology Narayana Health-Mazumdar Shaw Medical Centre, Bangalore. This work is licensed under Creative Commons Attribution-Noncommercial 4.0 International.

Professor Dr. B. K. Venkataraman's education in various schools in Mysore laid the groundwork for his academic pursuits. His academic determination propelled him to pursue higher education at Mysore Medical College, where he completed his medical training. Upon completing his studies at Mysore Medical College, he assumed the role of a lecturer, initially in anatomy and later in ophthalmology. He was transferred to Chikmagalur District Hospital as a registered medical officer (RMO). The Mysore Government was keen on establishing a dental college in Bangalore with Dr. S. Ramachandra as the first principal and deputed Dr. B.K. Venkataraman, along with a few others, to pursue a degree in dentistry at Nair Dental College, Bombay, in 1961. After successfully obtaining a Bachelor of Dental Surgery (BDS) degree, he returned as a faculty member at the recently established Government Dental College (GDC) in Bangalore, the first dental college in the state of Mysore, now Karnataka, marking a significant milestone in dental education. His involvement in planning the college, including the development of the BDS curriculum, was instrumental. Professor Dr. B.K. Venkataraman exhibited a steadfast commitment to the advancement of specialized domains within the discipline of dentistry [8]. His contributions were not only acknowledged but also propelled oral medicine into a crucial domain of education for dental practitioners [8]. His role in reintroducing the internship program for dentistry graduates was equally vital, addressing the need for hands-on clinical experiential learning and providing essential practical training for newly graduated dentists [8]. This experience was valuable in honing his skills in educational planning and curriculum design, which would be vital in his later efforts to introduce oral medicine as a subject at the undergraduate and postgraduate levels (Figure 2) [8].

**Figure 3:**
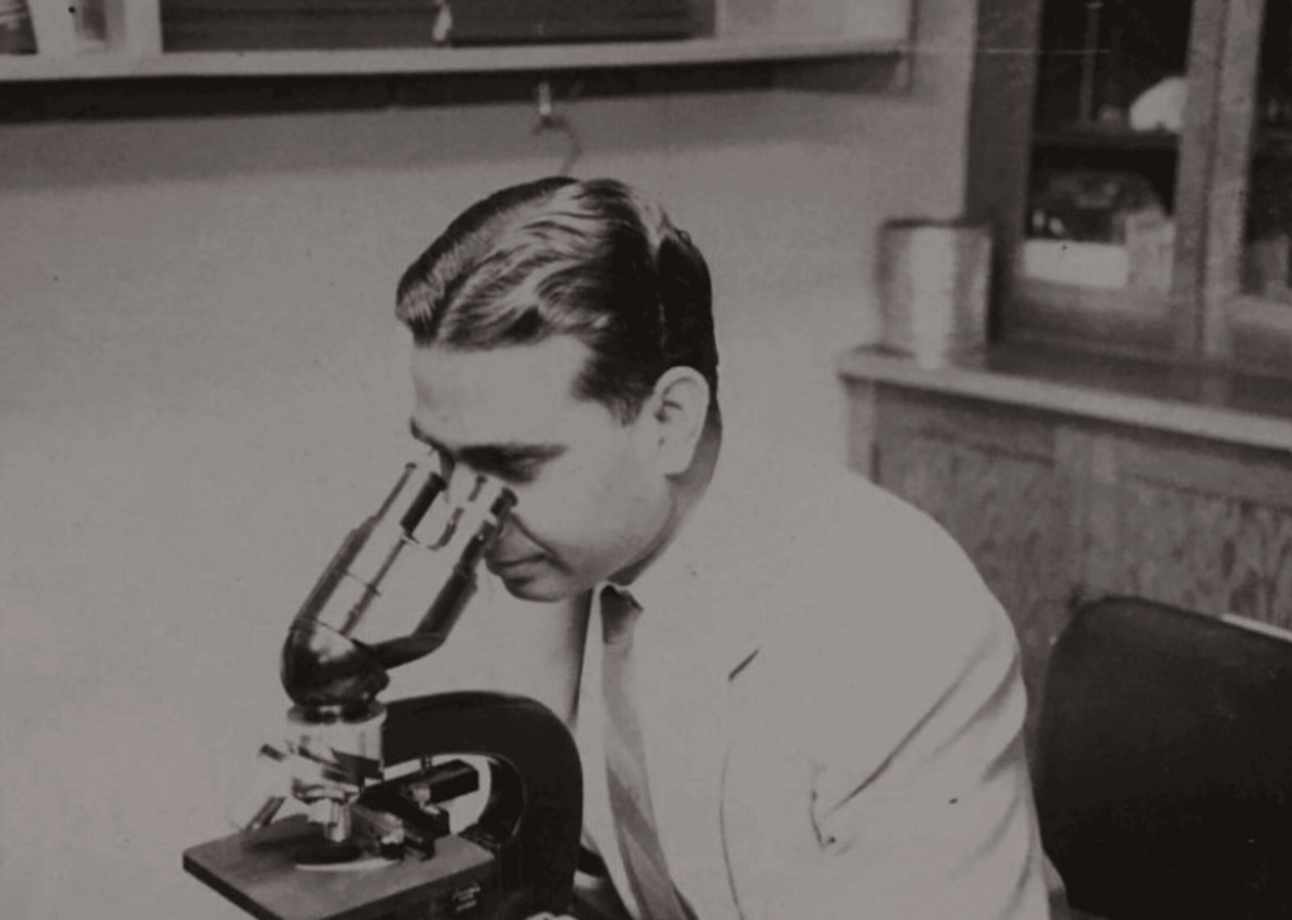
Professor Dr. B.K. Venkataraman at the pathology lab in the United States Image courtesy: Professor Dr. B.V. Murali Mohan, Senior Consultant and Physician, Academic Director in the Department of Internal Medicine and Pulmonology, Narayana Health-Mazumdar Shaw Medical Center, Bangalore. This work is licensed under Creative Commons Attribution-NonCommercial 4.0 International.

In 1963, Dr. B.K. Venkataraman was awarded a World Health Organization (WHO) fellowship to the United States of America (U.S.A.) [3]. He earned his Master of Science (M.Sc.) degree from the University of California and subsequently received extensive training in oral and maxillofacial surgery in London, West Germany, and Holland. Upon returning to Bangalore, he became a professor and vice principal at the GDC, Bangalore. The WHO chose Dr. B.K. Venkataraman to pursue further studies in oral medicine in 1970 at Edinburgh, where he was awarded a Fellowship in Dental Surgery by the Royal College of Surgeons of England (FDS-RCS). He returned to serve at the GDC in Bangalore and subsequently established the first department of oral medicine in the country (Figure 3) [4,5].

**Figure 4:**
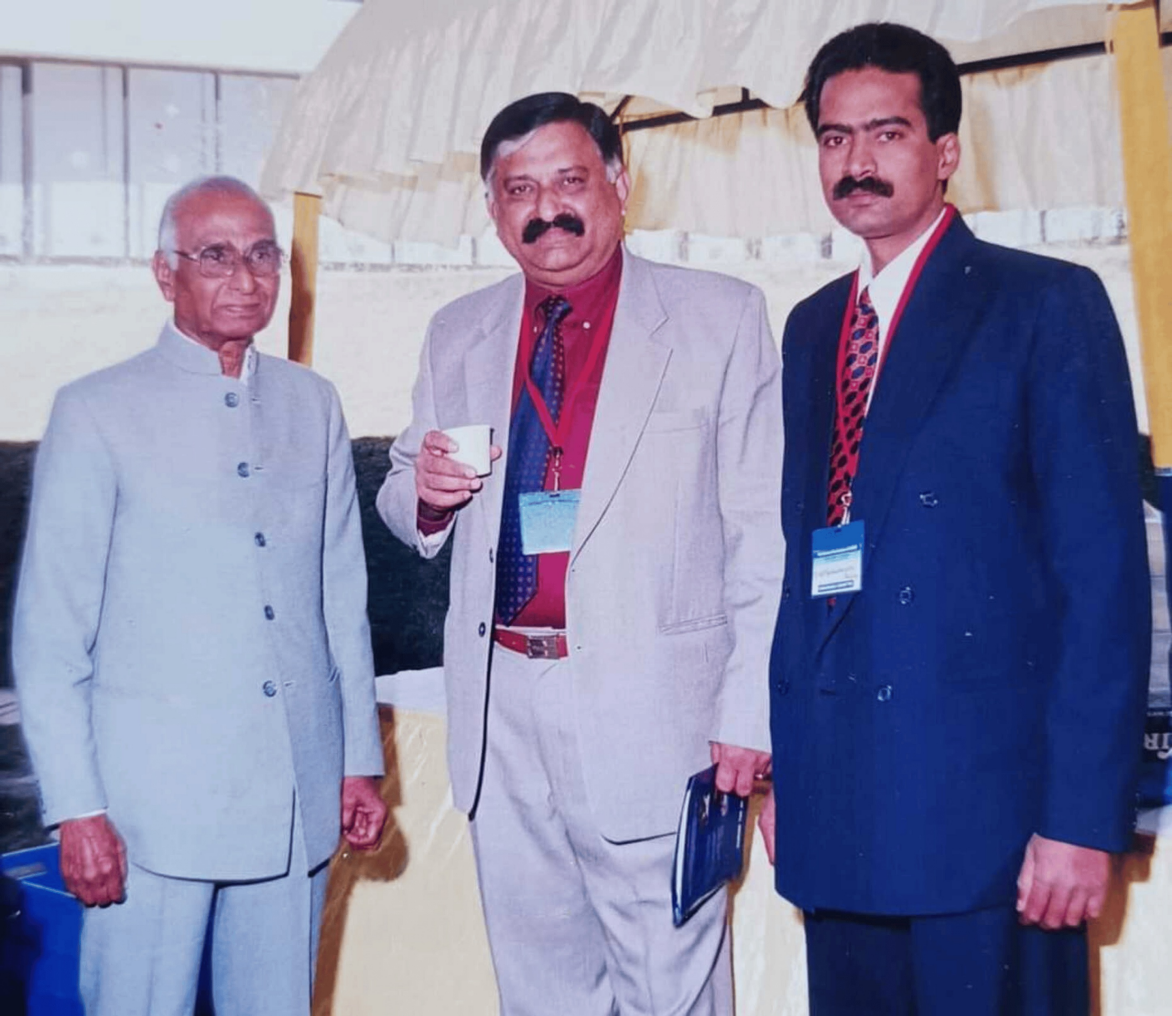
Professor Dr. B.K. Venkataraman, along with his old students, postgraduate student Dr. K.S. Ganapathy and Dr. G.V. Ramachandra Reddy, an undergraduate student at the National Oral Medicine Conference in Bangalore in 2002 Image courtesy: Professor. Dr. G.V. Ramachandra Reddy, Professor and Head of Department, Vinayaka Mission's Sankarachariyar Dental College, Vinayaka Mission's Research Foundation (Deemed to be University), Salem, Tamil Nadu, India. This work is licensed under Creative Commons Attribution-Noncommercial 4.0 International.

The GDC in Bangalore was the pioneering institution in India to introduce oral medicine as a specialized field of study for undergraduate and postgraduate programs. A passionate teacher, he was directly active in dental education for more than 38 years. In 1971, Dr. B.K. Venkataraman became the nation's first professor of oral medicine. Today, many of the senior professors in oral medicine in India are his former postgraduate students (Figure 4) [6, 7].

After the retirement of Dr. S Ramachandra, Dr. B.K. Venkataraman assumed the position of principal at the GDC, Bangalore, in 1974 and demonstrated his competence as a fitting successor to a distinguished individual. Having been elected to the Executive Committee of the Dental Council of India in 1974, he was chosen as the President of the Dental Council of India in the succeeding year. As the President of the Dental Council of India, he spearheaded the curriculum review for the BDS and Master of Dental Surgery (MDS) programs. From 1975 to 1978, he served as the Dean of the Faculty of Medicine at Bangalore University. During this time, he played a crucial role in initiating several new course offerings, including a Master of Chirurgiae (M.Ch.) in Pediatric Surgery and Urology, a Doctor of Medicine (M.D.) in Aviation and Ayurvedic Medicine, M.Sc. in Nursing, and the affiliation of Command Hospital with Bangalore University for (Doctor of Medicine) M.D. General Medicine and (Master of Science) and M.S. General Surgery Courses. Furthermore, he was crucial in initiating a bachelor's degree program in Unani medicine. Together with other department heads, he exerted an unwavering effort to enhance the infrastructure of the GDC Bangalore and uphold its impeccable educational standards [8].

After retiring from government services in 1979, he was invited to serve as professor of oral medicine and chairman of the oral medicine, radiology, and periodontia departments at the Faculty of Dentistry, Al-Arab Garyounis Medical University, Benghazi, Libya. Professor Dr. B.K. Venkataraman expressed his wish to resign from that post to return and serve in India. He acceded to the request of the Libyan Ministry of Health to return periodically as a visiting professor and examiner at that university until 1987 [7].

**Figure 5:**
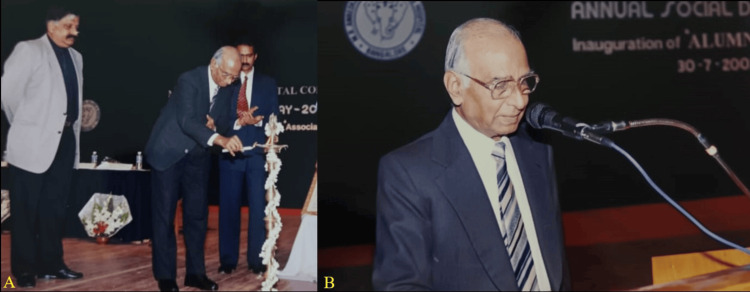
A) Professor Dr. B.K. Venkataraman lighting the lamp at the inauguration of the Alumini Association; B) Professor Dr. B.K. Venkataraman addressing the aluminis at Mathrusri Ramabai Ambedkar Dental College, Bangalore, on July 30, 2003. Image courtesy: Professor Dr. G.V. Ramachandra Reddy, Professor and Head of Department of Oral Medicine and Radiology, Vinayaka Mission's Sankarachariyar Dental College, Vinayaka Mission's Research Foundation (Deemed to be University), Salem, Tamil Nadu, India. This work is licensed under Creative Commons Attribution-Noncommercial 4.0 International.

The Ananda Social and Educational Trust was keen on starting a dental college and requested Dr. Venkataraman to assume the role of the founding principal of Mathrusri Ramabai Ambedkar Dental College, Bangalore. His efforts were instrumental in establishing Mathrusri Ramabai Ambedkar Dental College as the leading private-sector institution among the 20 dental colleges affiliated with Bangalore University. In 1992, he obtained authorization from the Dental Council of India for Mathrusri Ramabai Ambedkar Dental College to implement three postgraduate courses. He tendered his resignation from this position in November 1992 to pursue a life of retirement [8]. The management of Sri. Siddhartha Dental College, Tumkur, persuaded Dr. B.K. Venkataraman to assume control of the recently established institution in January 1993 (Figures 5A, 5B) [8].

Professional development

Pioneering Efforts

Dr. Bangalore Krishnaswamy Venkataraman's journey from humble beginnings to a leading figure in dentistry is a testament to his resilience and determination. His pioneering efforts in the field of oral medicine, despite the challenges he faced, serve as an inspiration to others in the field, encouraging them to pursue excellence despite obstacles [6].

Challenges Faced While Establishing Oral Medicine as an Undergraduate Subject

Professor Dr. B.K. Venkataraman faced several challenges while establishing oral medicine as an undergraduate subject at the GDC in Bangalore. These challenges, which included curriculum development, professional acceptance, and securing resources and support, were significant hurdles that he had to overcome in his efforts to formalize oral medicine as a subject [6, 7]. 

Curriculum Development

Professor Dr. B.K. Venkataraman aimed to develop a comprehensive curriculum that adequately covered the breadth of oral medicine. The curriculum development involved creating course materials, defining learning outcomes, and ensuring that the subject was integrated effectively into the existing dental education system [6,7].

Professional Acceptance

Professor Dr. Bangalore Krishnaswamy Venkataraman faced challenges while integrating oral medicine into the existing BDS curriculum. Gaining acceptance and recognition for oral medicine within the broader dental community was essential. Dr. B.K. Venkataraman had to work towards establishing the field's credibility, highlighting its relevance to overall dental practice and patient care. The initial reactions from the academic community to Dr. B.K. Venkataraman's introduction of oral medicine as an undergraduate subject was mixed, reflecting both support and skepticism [8]. Dr. B.K. Venkataraman also faced significant institutional resistance when he sought to establish oral medicine as a recognized undergraduate subject in dental education in India. Those who were accustomed to traditional dental curricula were initially skeptical about the need for a dedicated subject of oral medicine, questioning its relevance and the need for additional resources [8]. Others questioned how it would fit the current structure, the adequacy of time allocated for this new discipline, and whether it would overlap with other subjects. However, several fellow academicians recognized the necessity of oral medicine as a distinct field within dentistry. They acknowledged that a focused curriculum would enhance the training of dental students in diagnosing and managing oral diseases, thus improving patient care [8].

Securing Resources and Support

The logistical hurdle Professor Dr. B.K. Venkataraman had to overcome the requirement for obtaining the necessary funding and resources from the institution for establishing a new subject area, which required additional resources, such as qualified faculty, teaching materials, and facilities for practical training [8].

## Review

Major milestones

*Establishment of the First Dental College at Bangalore* *in Karnataka State*

In 1958, Professor Dr. Bangalore Krishnaswamy Venkataraman was selected by the Government of Mysore, alongside Dr. S. Ramachandra, to plan the establishment of the GDC at Bangalore. It was the first dental college in the Mysore district in Karnataka state, marking a significant milestone in dental education. His involvement in planning the college included developing the BDS curriculum, which laid the groundwork for dental education in India. This experience honed his educational planning and curriculum design skills, which would be instrumental in his later efforts to formalize oral medicine as a subject [9].

Establishment of Oral Medicine as a Discipline

Professor Dr. B.K. Venkataraman's initiative to introduce oral medicine as a subject in undergraduate dental education at GDC in Bangalore had a profound impact on the global dental community. His efforts have not only formalized the training and education of dental students in diagnosing and managing oral diseases but also set a new standard for dental education. This initiative has inspired a modern curriculum within Indian dental schools and set a precedent for other countries to recognize the importance of oral medicine as a distinct field. His pioneering work has encouraged dental schools worldwide to incorporate similar courses, thereby broadening the scope of dental education globally and fostering a sense of pride and unity among dental educators, practitioners, and students [9, 10].

President of the Dental Council of India

Professor Dr. B.K. Venkataraman served as the president of the Dental Council of India from October 14, 1976, to September 3, 1978 [11]. One of his notable accomplishments in this position was the commencement of a thorough reform of the curricula for the BDS and MDS programs. The primary objective of this endeavor was to augment the quality of dentistry education, guaranteeing its alignment with global benchmarks and its ability to equip students for their future careers in the field sufficiently. During his tenure as President, the Dental Council of India prioritized the preservation of elevated benchmarks in dental education and professional conduct. The activities mentioned above encompass the performance of inspections on dental schools and the enforcement of specified criteria, contributing to the enhancement of the general standard of dental institutions nationwide. Professor Dr. B.K. Venkataraman exerted a significant and enduring influence on dental policy in India, thereby shaping the legislation that governs dental practice and education [11, 12].

Founder of Professional Organization, the Indian Academy of Oral Medicine and Radiology

**Figure 6:**
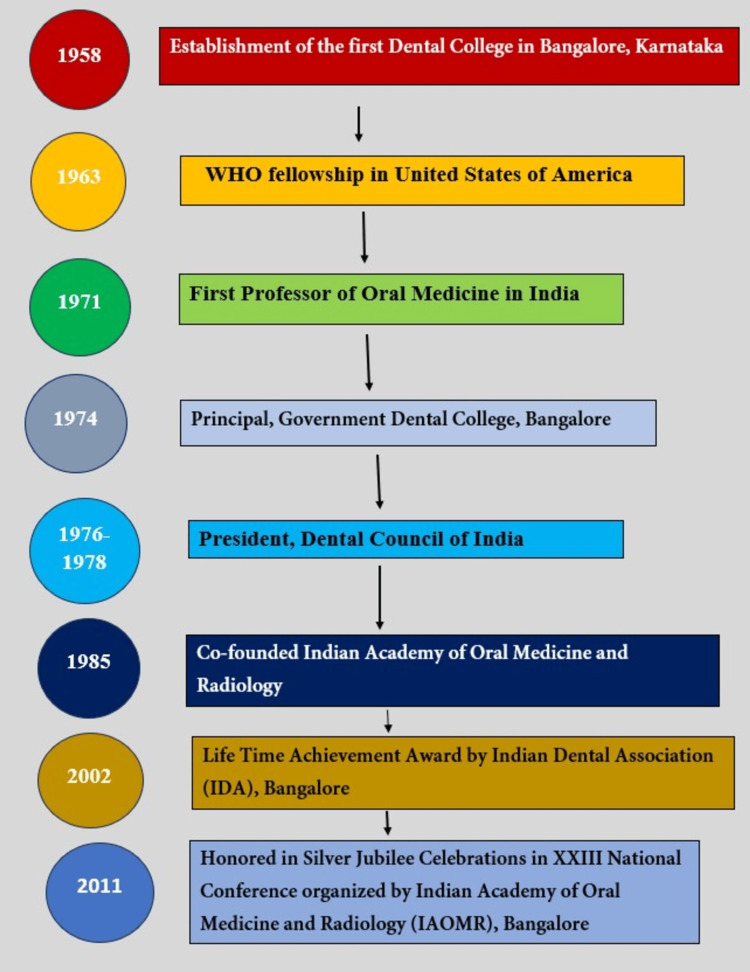
A brief historical overview or timeline of history of major milestones of Professor Dr. Bangalore Krishnaswamy Venkataraman This image has been created by the author. This work is licensed under Creative Commons Attribution-Noncommercial 4.0 International.

Professor Dr. B.K. Venkataraman was crucial in the establishment of the Indian Academy of Oral Medicine and Radiology. In 1985, he co-founded the Indian Academy of Oral Medicine and Radiology, an organization with a global vision to promote the specialty, enhance educational standards, facilitate research in oral medicine, and foster a community of professionals dedicated to advancing the specialty in India. Under his leadership, this academy has not only significantly influenced dental education in India but also made a mark globally [10]. The Academy now has a membership of more than 3,050 life and associate members. It operates three state branches nationwide and coordinates national-level conferences, undergraduate and postgraduate conventions, and the Triple O Symposium annually (Figure 6) [13].

Advocacy for Research and Development

Professor Dr. B.K. Venkataraman advocated for the importance of research in oral medicine throughout his career. His emphasis on evidence-based practices encouraged dental schools to prioritize research initiatives, leading to advancements in diagnostic techniques and treatment options that had a global impact [14].

Research and Innovation

Professor Dr. B.K. Venkataraman was passionate about encouraging research. His research support has been transformative for dental education. He supported Professor C.V. Mohan in initiating a (Doctor of Philosophy) PhD Program in oral medicine and radiology in 2001 under Dr. B.K. Venkataraman Education Society for Higher Education in Dental Sciences. This society is associated with and recognized by the Rajiv Gandhi University of Health Sciences. His emphasis on the importance of research in oral medicine has encouraged dental schools to prioritize research initiatives, leading to significant advancements in diagnostic techniques and treatment options. This transformative focus on research had a ripple effect, influencing curricula in other countries to include research methodologies and evidence-based practices in dental education [14].

International Global Collaboration and Knowledge Exchange

Professor Dr. B.K. Venkataraman, enriched by his international experiences at the University of California and Edinburgh, brought a global perspective on diagnostic methods to India. His work has not only fostered international collaboration and knowledge exchange among dental professionals but also significantly enriched dental education and practice worldwide [15].

Legacy of Excellence

Professor Dr. B.K. Venkataraman's legacy is marked by his unwavering commitment to improving oral health care in India, and his contributions continue to impact the field and inspire future generations of dental professionals. His role in advancing dental education and comprehensive oral health care is of utmost importance, as it has left an indelible mark on global dental education. His commitment to improving dental education has encouraged ongoing developments in curricula and training programs that prioritize comprehensive oral health care. Through these contributions, Professor Dr. B.K. Venkataraman has played a crucial role, in ensuring that oral medicine is recognized as a vital component of comprehensive dental care [15].

Awards and honorariums

Professor Dr. Bangalore Krishnaswamy Venkataraman received several honors and awards throughout his distinguished career, recognizing his contributions to dentistry and oral medicine [16]. Key accolades of Dr. B.K. Venkataraman are listed below.

Lifetime Achievement Award

In 2002, Professor Dr. B.K. Venkataraman was bestowed with the prestigious Lifetime Achievement Award, a testament to his dedicated and outstanding service to the field of dentistry in Karnataka. This recognition, presented by the esteemed Indian Dental Association, Bangalore, underscores the significance of his contributions over more than 40 years in the profession, a period during which he has significantly advanced the field [16, 17].

Honors from the Indian Academy of Oral Medicine and Radiology

**Figure 7:**
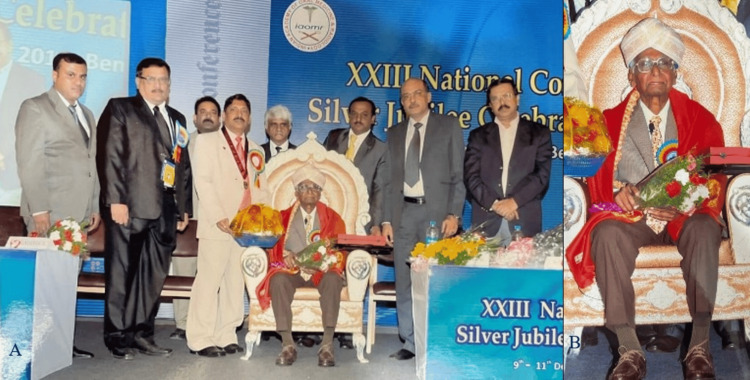
A) Professor Dr. Bangalore Krishnaswamy Venkataraman was felicitated for his contribution to the specialty during the XXIII National Silver Jubilee Conference of the Indian Academy of Oral Medicine and Radiology on December 9, 2011, in Bangalore ; B) Professor Dr. Bangalore Krishnaswamy Venkataraman Image courtesy: Dr. T.K. Ramamurthy, Professor and Head, Department of Oral Medicine and Radiology, Vydehi Institute of Dental Sciences and Research Centre, Whitefield, Bangalore. This work is licensed under Creative Commons Attribution-Noncommercial 4.0 International.

During the XXIII National Conference, 2011, the Indian Academy of Oral Medicine and Radiology Association celebrated the silver jubilee by honoring esteemed founder member Dr. B. K. Venkataraman for his outstanding contributions to the specialty. His dedication and vision for oral medicine and radiology were instrumental in shaping the journey over the past 25 years (Figures 7A, 7B) [18]. ​​​​

He was hailed as a "perfect teacher" who not only understood students' problems but also significantly boosted their self-esteem. His exceptional intelligence, ability to explain complex concepts, and unwavering passion for teaching left a profound impact on his students, earning their deep appreciation and respect [19, 20].

Innovations 

Professor Dr. Bangalore Krishnaswamy Venkataraman introduced several key innovations in his approach to diagnosing oral diseases, significantly advancing the field of oral medicine and pathology in India [20]. His notable contributions are mentioned below.

Intralesional Corticosteroid Injections

Professor Dr. B.K. Venkataraman pioneered the infusion of hydrocortisone sodium succinate into the submucosal cavity in 1969 to treat oral submucous fibrosis [21]. Professor Dr. B.K. Venkataraman and visiting WHO professor Joseph Echeler from West Germany played a key role in the management of oral submucous fibrosis [21].

Orthopantomograph X-Ray Studies

In 1973, Professor Dr. B.K. Venkataraman conducted a comprehensive study of 1,500 orthopantomography X-ray images, which was supported by the Indian Council of Medical Research (ICMR). This research established protocols for interpreting normal anatomical structures and identifying pathologies in the jaw and oral cavity, enhancing diagnostic accuracy in dental practice [21].

Advocacy for Advanced Diagnostic Techniques

Professor Dr. B.K. Venkataraman, through his training and experiences, including his time at the University of California and the University of Edinburgh, brought international perspectives on diagnostic methods to India. He advocated for the integration of advanced techniques in imaging and immunohistochemistry into routine diagnostic practices in oral pathology [21].

Research Publications and Professional Legacy

**Figure 8:**
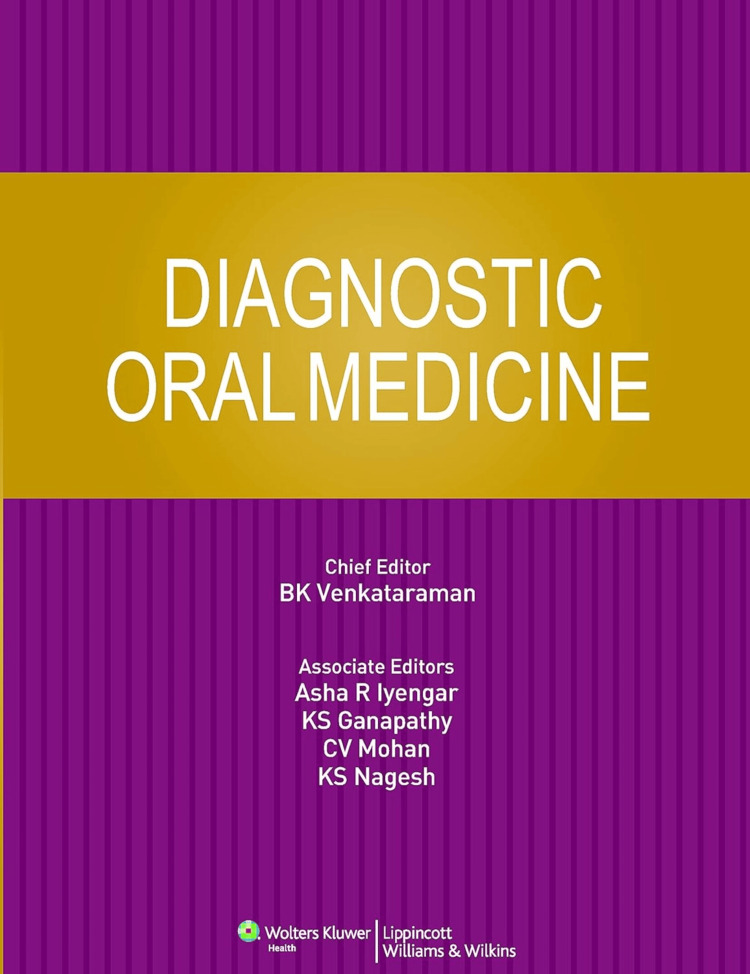
Textbook  by Professor Dr. B.K. Venkataraman titled "Diagnostic Oral Medicine" Image Source: [22] This work is licensed under Creative Commons Attribution-Noncommercial 4.0 International.

Professor Dr. B.K. Venkataraman, in collaboration with Dr. J.C. Southam, has authored a publication titled "Oral manifestations of leprosy" [21]. Professor Dr. B.K. Venkataraman was the chief editor of the multi-author textbook "Diagnostic Oral Medicine," which provides a detailed examination of the diagnosis and management of oral disorders [22]. The book, uniquely structured to cover various aspects such as etiology, pathophysiology, clinical features, and treatment options, is a vital resource for students and practitioners. It encapsulates the legacy of Professor Dr. B.K. Venkataraman, a great educator known for his commitment to teaching and mentorship. His influence, reflected in the methodologies and insights into oral health care, continues to inspire dental schools and practitioners (Figure 8) [22].

**Figure 9:**
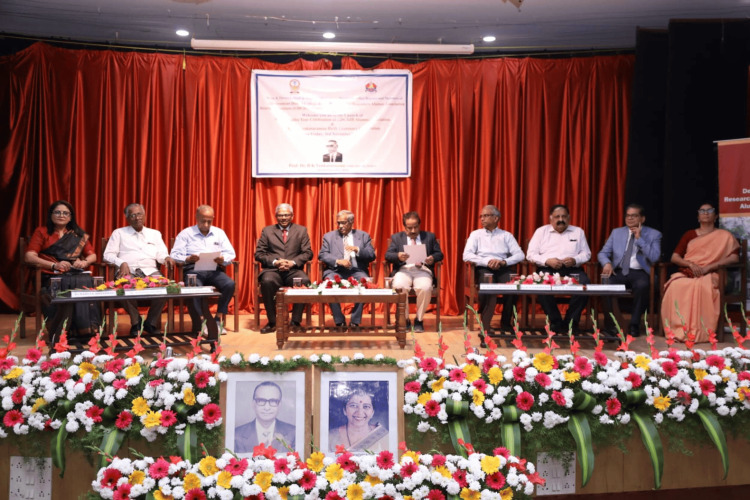
Launch of the Dr. B.K. Venkataraman Birth Centenary celebration in association with the Government Dental College Research Institute Bangalore (GDCRIB) Alumini Association on November 3, 2023 Image courtesy: Dr. K.R. Vijayalakshmi, Professor and Head of Oral Medicine and Radiology, Government Dental College, Bangalore. This work is licensed under Creative Commons Attribution-Noncommercial 4.0 International.

In commemoration of the centenary birthday celebration of Professor Dr. B.K. Venkataraman, the centenary committee, and the Government Dental College and Research Institute, Bangalore (GDCRIB) Alumni Association have organized several continuing dental education programs (Figure 9).

These programs, held at Government Dental College, Bangalore, Rajarajeshwari Dental College, Rashtreeya Vidyalaya (RV) Dental College, Mathrusri Ramabhai Ambedkar Dental College, Bangalore, and Sri Siddhartha Dental College and Hospitals, Tumkur, in association with the Indian Academy of Oral Medicine and Radiology-Karnataka state branch, offer a wealth of educational insights for all those in the dental field. Following these programs, a valedictory function will be organized on October 26, 2024.

## Conclusions

Professor Dr. Bangalore Krishnaswamy Venkataraman, a distinguished professor in the field of oral medicine, is widely recognized for his expertise. His academic and clinical contributions to oral medicine are highly impactful. With a career spanning several decades, he has been instrumental in advancing research, improving diagnostic techniques, and enhancing patient care in oral medicine. His work is not only recognized nationally but also internationally, and he has been involved in numerous research projects, publications, and conferences. His dedication to teaching and mentoring the next generation of dental professionals has earned him respect and admiration.

In summary, Professor Dr. B.K. Venkataraman's work in oral medicine has significantly contributed to the field, making him a prominent figure whose influence will continue to be felt for years. His life and work reflect a deep dedication to oral medicine, making him a revered figure in Indian dentistry. His contributions have not only advanced clinical practices but also inspired future generations to pursue excellence in oral health care, ensuring a lasting impact on the field.
